# Annotations

**Published:** 1952-01

**Authors:** 


### *Annotations*

### REORGANIZATION OF THE AMBULANCE SERVICE

The 1946 Act, while removing many of the functions of the Local Authority in the treatment of patients, placed on them responsibility for organizing a comprehensive ambulance service. In Bristol this was placed in the hands of the Chief Fire Officer and was integrated with the Fire Service. The efficiency of the organization created has earned widespread approval. In the first quarter after the appointed day 11,000 calls were made, and in the equivalent quarter of 1951 the number had reached 30,000.

The revival of the Civil Defence Service has led the Minister of Health to decide on the separation of the Fire Service and the Ambulance Service in the event of an emergency. The Bristol City Council decided that such a separation could not be carried out easily when an emergency arose, and decided to make the change now.

Amending proposals under the National Health Service Act are now before the Minister of Health to enable this change to take place. It is intended that the Ambulance Service will be placed under the control of the Medical Officer of Health. The central control will be based in the Central Health Clinic and the Ambulance Stations will be situated at a Central Depot, Brislington, Fishponds and Feeder Road, to cover the needs of the City. Contact points for the Ambulance drivers will also be established at Clinics throughout the City. The change-over is likely to take place early in the New Year.

The Council is also considering the introduction of a radio-telephone system to link ambulances with their headquarters. This is already in use in America and in Cardiff and experiments show that it avoids the need to build expensive ambulance depots throughout the area and saves many empty journeys.

### HOME NURSING EQUIPMENT

There are many patients occupying hospital beds who could well be nursed at home by their families or by the District Nursing Association under the guidance of the patient's own doctor, if the necessary equipment were available. The loan of such equipment has been undertaken on a limited scale in the past by the District Nursing Association, the B.R.C.S., St. John's Ambulance Brigade and the Tuberculosis Voluntary Care Committee but the demand has outrun the supply, and there are great difficulties in its delivery, collection and sterilization.

The Health Committee of the Bristol City Council is arranging to provide a comprehensive loan service for home nursing equipment under the care of the Medical Officer of Health, to be available at the request of a practitioner, district nurse or midwife, starting on 1st January, 1952. A central equipment store at Feeder Road is nearing completion and is placed beside the Disinfecting Station. Stocks of the smaller items of equipment will be maintained at nine district clinics and five district nurses hostels, and a collection service will distribute to and collect from these centres. Except in necessitous cases a cash deposit of ten per cent of the cost of the equipment borrowed will be asked to ensure its safe return. The capital expenditure on this plan is likely to be £7,000 and it is estimated £2,500 a year will be required for its maintenance. The Health Committee hope to expand the service later to include a syringe service, but the cost of such a service is very high and it seems doubtful whether the necessary funds will be available for some years.

### CLINICAL MEETINGS

The best way of running clinical meetings has been under discussion recently, and, in order to meet the wishes of as many practitioners as possible, a sub-committee was appointed last summer to consider the whole problem. It had representatives from the Medical Committee of the United Bristol Hospitals, the Bristol Medico-Chirurgical Society and the Bristol division B.M.A. and met with Dr. Beryl Corner in the chair.

The recommendations of this sub-committee have now been accepted by the appointing groups. Two meetings a year are planned, one held by the Medico-Chirurgical Society in November and the other in the summer to be run by the B.M.A. It is proposed to try having a combined type of meeting in which patients are available for examination from 8 p.m. to 9.30 p.m. and an hour's discussion of the cases should follow. It was hoped that twelve groups of cases would be available and each would include several patients with a similar condition to make their examination by doctors easier.

The November clinical meeting at Frenchay Hospital was organized with these recommendations in mind and it seems to have obtained widespread approval. A report on that meeting appears elsewhere in this issue.

### BRISTOL MATERNITY FLYING SQUAD

On 1st January, 1952, a Maternity Flying Squad became available at Southmead Hospital under the direction of Professor Lennon to assist practitioners in the Bristol clinical area faced with obstetrical emergencies.

The team will consist of a Hospital Obstetrical Officer and a Midwifery Sister travelling in a car equipped with sterilized emergency obstetrical and transfusion equipment. The squad is available twenty-four hours a day and aims to be on the road within ten minutes of being called.

The commonest emergency which is anticipated is post-partum haemorrhage. All patients in the Bristol clinical area are entitled to the help of the Flying Squad, whatever obstetrical arrangements have been made, and its services may be enlisted by telephoning Southmead Hospital.

